# Association between MEG3/miR-181b polymorphisms and risk of ischemic stroke

**DOI:** 10.1186/s12944-018-0941-z

**Published:** 2018-12-22

**Authors:** Xuemei Han, Zhaoshi Zheng, Chunhui Wang, Libo Wang

**Affiliations:** 1 No. 1 Department of Neurology, China-Japan Union Hospital of Jilin University, Changchun, Jilin 130031 People’s Republic of China; 2 Department of Neurosurgery, the Hospital of Jilin Province, Changchun, Jilin 130031 People’s Republic of China

**Keywords:** Long non-coding RNAs, Maternally expressed gene 3, miR-181, Polymorphism, Ischemic stroke

## Abstract

**Background:**

Recent evidence suggests that long non-coding RNAs (lncRNAs) are key regulators in the pathological process of ischemic stroke (IS). Maternally expressed gene 3 (MEG3) was observed to be up-regulated in IS, acting as a competing endogenous RNA for miR-181b to regulate ischemic brain injury. The purpose of this study was to evaluate the association of tagSNPs in MEG3 (i.e., rs7158663 and rs4081134) and miR-181b rs322931 with IS risk.

**Methods:**

Genomic DNA was extracted from blood samples of 509 patients with IS and 668 healthy controls. Genotyping of MEG3 rs7158663, rs4081134, and miR-181b rs322931 was performed by TaqMan assay. The transcriptional activity was measured using the Dual-Luciferase Reporter Assay kit.

**Results:**

Single-site analysis revealed a significantly higher risk of IS being associated with miR-181b rs322931 CT and CT/TT genotypes (CT vs. CC: adjusted OR = 1.48, 95% CI: 1.13–1.95, *P* = 0.005; CT/TT vs. CC: adjusted OR = 1.52, 95% CI: 1.17–1.97, *P* = 0.002). Combined analyses revealed that combined genotypes (rs7158663 GG + rs322931 CT/TT and rs7158663 AG/AA + rs322931 CT/TT) increased IS risk compared to genotypes of rs7158663 GG + rs322931 CC. Stratification analyses showed that patients carrying miR-181b rs322931 CT/TT genotypes had higher levels of low-density lipoprotein cholesterol (LDL_C) (*P* = 0.01). Moreover, results from logistic regression analysis showed that rs322931 CT/TT genotypes were risk factors besides hypertension, total cholesterol, triglyceride, and LDL_C. Further dual-luciferase reporter assay showed that the rs322931 T allele had lower levels of luciferase activity than the rs322931 C allele.

**Conclusion:**

These findings indicate that miR-181b rs322931 may singly or jointly contribute to the risk of IS.

## Introduction

Stroke is the major cause of morbidity and mortality worldwide, with about 15 million new cases and 5 million death each year [1, 2]. Ischemic stroke (IS), the most common type, occupies 85–90% of total strokes [3, 4]. China, the most populous country in the world, is facing serious public health implications of IS epidemic [5–7]. Etiologically, IS is a complex disorder, involving in a series of risk factors including hypertension, dyslipidemia, obesity, diabetes, smoking as well as genetic factor [8–11]. Adults with both high triglyceride (TG), low high-density lipoprotein cholesterol (HDL-C), and high low-density lipoprotein cholesterol (LDL-C), particularly those with diabetes, have an increased risk of IS [12]. Supplementation with plant extracts may result in improvement of glycemic and serum lipid levels in patients with dyslipidemia, and finally reduce the risk of IS [13, 14]. Genetic dissection of IS has targeted many association studies and identified some potential candidate genes such as apolipoprotein A-V, apolipoprotein B, ATP-binding cassette transporter 1, brain-derived neurotrophic factor (*BDNF*), and arachidonate 5-lipoxygenase-activating protein (*ALOX5AP*) [15–17]. The selection of these genes was based on important signal pathways in the process of pathophysiology of IS, such as apolipoprotein homeostasis, neurological injury recovery, inflammation and abnormalities in 5-lipoxygenase metabolism [15–17]. Nevertheless, there are several other functional candidate genes that are of great value to be analyzed.

Non-coding RNAs were initially thought to be transcriptional noise without any function. However, it is nowadays believed to play crucial roles in the regulation of information flow from DNA to protein, such as DNA replication, RNA splicing, and translation regulation [18–20]. According to the length, non-coding RNAs were classified into microRNAs (miRNAs, 18–24 nucleotides), long non-coding RNAs (lncRNAs, ≥ 200 nucleotides) and etc. Recently, amounts of non-coding RNAs were found to be aberrantly expressed in IS [21–25]. Among them, maternally expressed gene 3 (MEG3) was observed to be up-regulated in IS, acting as a competing endogenous RNA for miR-181b [26–29]. Knockdown of MEG3 can protect against ischemic damage and improve neurobehavioral outcomes [26, 30]. These findings indicate that the abnormal expression of MEG3 may be a key event in the pathophysiology of IS.

Currently, miRNAs/lncRNAs-related single nucleotide polymorphisms (SNPs) have been demonstrated to be predictors of susceptibility of IS by influencing their expression [31, 32]. In 2016, Cao et al. reported a tagSNP in MEG3 (rs7158663) and the rs7158663 AA genotype had a 96% increased risk of colorectal cancer [33]. In 2018, Zhuo et al. reported another tagSNP in MEG3 (rs4081134) and carriers with rs4081134 AG/AA genotypes tended to develop neuroblastoma among children > 18 month of age and clinical stage III/IV disease [34]. Moreover, a genetic variant of rs322931 was reported to influence the expression of miR-181a and miR-181b [35]. To date, no association study was conducted for the 3 SNPs with IS risk. In this study, we instigated the association between the 3 SNPs and IS susceptibility in a Chinese Han population comprising 509 IS patients and 668 age-, gender-, and ethnicity-matched controls. Our purpose was to identify new candidates for the etiology of IS.

## Materials and methods

### Study population

The study population was described in our previous study [36]. Briefly, a total of 509 patients with IS and 668 controls were consecutively recruited from the China-Japan Union Hospital of Jilin University between March 2014 and July 2017. Diagnosis of IS was made according to clinical manifestations and subsequently confirmed by computed tomography scans and magnetic resonance imaging. Patients were excluded if they had hemorrhagic stroke, subarachnoid hemorrhage, traumatic brain injury, malignancy, or other brain inflammatory diseases. IS subtypes were classified according to the criteria of Trial of Org 10,172 in Acute Stroke Treatment as described previously [37]. Controls were healthy subjects from the same hospital during the same time period as the cases. The controls were frequency matched to cases with regard to age, gender, living area, and ethnicity. The exclusion criteria for controls were: i) non-Chinese Han population; ii) having a family history of IS. Diagnosis of hypertension was made according to the criteria: blood pressure ≥ 140/90 mmHg on two consecutive occasions at least 24 h apart or receiving antihypertensive therapy [38]. Diagnosis of diabetes mellitus was made according to the criteria: two fasting glucose ≥7.0 mmol/L and 2 h postload glucose ≥11.1 mmol/L or receiving treatment with hypoglycemic drugs. Fast serum levels of total cholesterol (TCH), TG, HDL-C, and LDL-C were taken from the hospital database system. The study protocol was approved by the Institutional Ethnical Committee of the China-Japan Union Hospital of Jilin University, and informed consent was signed by each subject. After informed consent was obtained, all subjects contributed 3–5 ml peripheral venous blood.

### SNPs selection

**Fig. 1:**
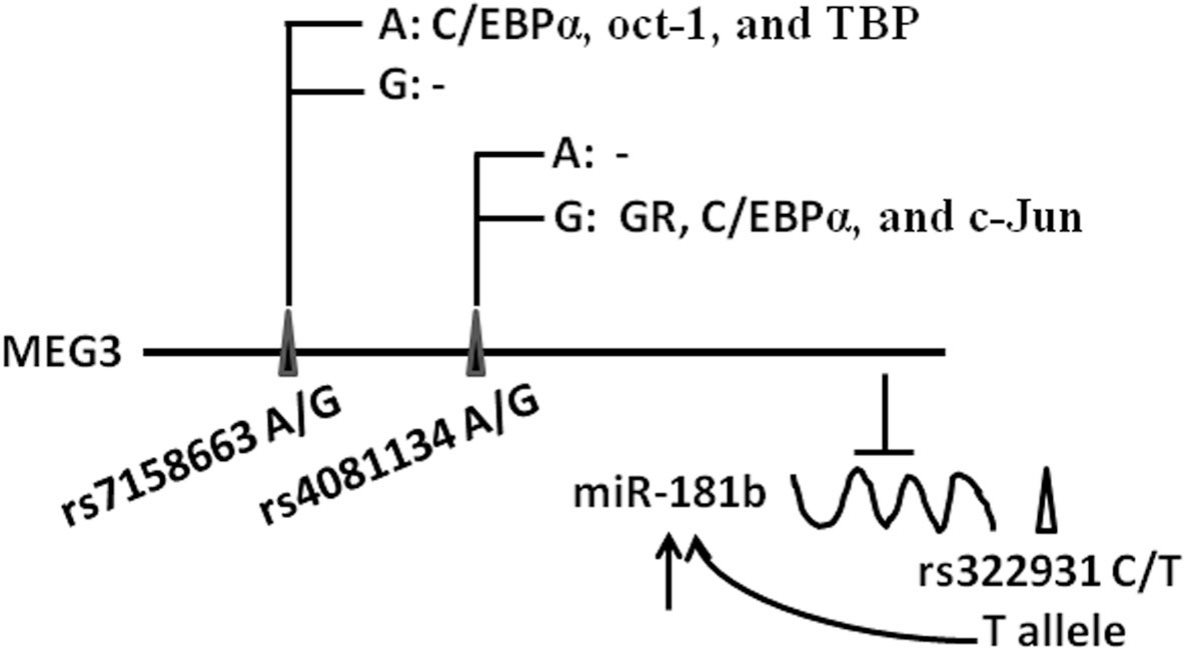
SNPs related to lncRNA MEG3 and miR-181b. ▲ TagSNPs in MEG3 (i.e., rs7158663 and rs4081134). In silica analysis showed that the rs7158663 A can bind to transcriptional factors [C/EBPα, oct-1, and TATA bingding protein (TBP)] and the rs4081134 G can bind to transcriptional factors [(glucocorticoid receptor GR), C/EBPα, and c-Jun], whereas the rs7158663 G and rs4081134 A can not. ∆ Genome-wide association study indentified a functional polymorphism rs322931. The T allele exhibited higher levels of miR-181b

We selected SNPs according to the following criteria: i) tagSNPs of MEG3; ii) minor allele frequency > 0.10 in Han Chinese from the 1000 Genome Projects; and iii) in silica analysis predicted that SNPs located in the binding sites of transcription factors. Finally, two MEG polymorphisms of rs7158663 G > A and rs4081134 G > A were selected for analysis in this study. Moreover, we selected a functional SNP of rs322931 that affects the expression of miR-181a and miR-181b in human brain and blood [35]. The detailed information of the 3 SNPs is described in Fig. 1.

### DNA extraction and genotyping

Genomic DNA was extracted from blood samples using the Genomic DNA Isolation Kit (Tiangen, Beijing, China). SNPs genotyping was performed by TaqMan assay on an ABI 7500 real-time PCR System (Applied Biosystems, Foster City, CA, USA). The probe ID for rs7158663, rs4081134, and rs322931 was C__9693465_10, C__1259786_10, and C__26961572_20, respectively. For quality control, a negative control of distilled water without template was used in each 96-well plate. Furthermore, about 5% randomly selected samples were reanalyzed by Sanger sequencing, and the results were completely identical.

### Dual-luciferase activity assay

Luciferase activity was detected as previously described [36]. Briefly, 2 fragments were amplified using DNA template from two individuals homozygous for either the rs322931 CC or rs322931 TT genotype. The products were cloned into the pGL3 vector containing a firefly luciferase reporter gene (Promega, Madison, WI, USA). The vectors were transiently transfected into cultured HEK293 cells using the Lipofectamine 3000 (Thermo Fisher Scientific, Waltham, MA, USA), and transcriptional activity was measured using the Dual-Luciferase Reporter Assay kit (Promega) at 48 h after transfection. The pRL-TK vector with a renilla luciferase reporter gene (Promega) was co-transfected for normalization. The relative luciferase activity was reported as the ratio of firefly to luciferase activity.

### Statistical analysis

Statistical data was analyzed using the SPSS software version 19.0 (SPSS, Chicago, IL, USA). Continuous data was presented as mean ± standard deviation and compared using the Student’s *t*-test. Categorical data was presented as number (proportions) and compared using chi-squared test. Hardy-Weinberg equilibrium (HWE) for each SNP in both cases and controls were compared using χ ^2^ test. The associations of single and combined genotype analyses with IS risk were evaluated using odds ratios (ORs) and 95% confidence intervals (CIs) after adjustment of age, gender, hypertension, and diabetes mellitus. The linkage disequilibrium (LD) and the association of haplotypes with risk of IS were estimated by the SHEsis software [39]. For multiple comparison, *P* <  0.017 (0.05/3) was considered as statistically significant after Bonferroni’s correction. Multivariable logistic regression analysis was performed to exclude potential confounding variables with age, gender, hypertension, diabetes mellitus, TCH, TG, HDL-C, LDL-C, MEG3 rs7158663, rs4081134 and miR-181b rs322931 entered as covariates in the model. *P* values less than 0.05 were considered as significant.

## Results

### Baseline characteristics of the study population

The baseline features of the study population are presented in Table 1. The mean age of cases and controls was 59.9 ± 10.9 and 58.6 ± 12.2, respectively. There were 327 males and 182 females in cases and 414 males and 254 females in controls. No significant difference was observed between the 2 groups for age and gender (*P* > 0.05). Compared with controls, patients with IS had a higher prevalence of hypertension and diabetes mellitus and higher levels of TCH, TG, and LDL-C.

**Table 1:** Characteristics of the study population

Variables	Controls, *n* = 668	Patients with IS, *n* = 509	*P* value
Age, mean (± SD)	58.6 (± 12.2)	59.9 (± 10.9)	0.06
Gender (%)			
Male	414 (62.0)	327 (64.2)	0.43
Female	254 (38.0)	182 (35.8)	
Hypertension, n (%)			
Yes	132 (19.8)	280 (55.0)	< 0.001
No	536 (80.2)	229 (45.0)	
Diabetes mellitus, n (%)			
Yes	72 (10.8)	80 (15.7)	0.01
No	596 (89.2)	429 (84.3)	
TCH, mmol/L	4.68 ± 0.79	5.04 ± 0.72	< 0.001
TG, mmol/L	1.11 ± 0.36	1.84 ± 1.10	< 0.001
HDL-C, mmol/L	1.56 ± 0.36	1.57 ± 0.38	0.53
LDL-C, mmol/L	2.26 ± 0.97	2.69 ± 0.98	< 0.001

*IS* ischemic stroke, *SD* standard deviation; TCH, total cholesterol, *TG* triglyceride, *HDL-C* high-density lipoprotein cholesterol, *LDL-C* low-density lipoprotein cholesterol

### Association between polymorphisms in MEG3/miR-181b and IS risk

The genotype frequencies of MEG3 rs7158663, rs4081134 and miR-181b rs322931 were in HWE in both cases and controls (*P* > 0.05). miR-181b rs322931 CT and CT/TT genotypes were more frequent in IS patients than in controls (33.4 vs. 26.8% and 38.1 vs. 29.2%). A significantly higher risk of IS was associated with rs322931 CT and CT/TT genotypes after adjusting to age, gender, hypertension, and diabetes mellitus (CT vs. CC: adjusted OR = 1.48, 95% CI: 1.13–1.95, *P* = 0.005; CT/TT vs. CC: adjusted OR = 1.52, 95% CI: 1.17–1.97, *P* = 0.002). However, no evidence of association was observed between MEG3 rs7158663 and rs4081134 and IS risk (Table 2).

**Table 2:** Association between polymorphisms in MEG3/miR-181b and risk of ischemic stroke

Polymorphism	Controls, *n* = 668 (%)	IS, *n* = 509 (%)	Crude OR (95% CI)	*P* value	Adjusted OR(95% CI)^†^	Adjusted *P* value^†^
MEG3 rs7158663						
GG	372 (55.7)	252 (49.5)	1.00		1.00	
AG	242 (36.2)	202 (39.7)	1.23 (0.96–1.58)	0.10	1.19 (0.92–1.55)	0.19
AA	54 (8.1)	55 (10.8)	1.50 (1.00–2.26)	0.05	1.49 (0.96–2.32)	0.08
Dominant model	296 (44.3)	257 (50.5)	1.28 (1.02–1.62)	0.04	1.25 (0.98–1.60)	0.08
Recessive model	614 (91.9)	454 (89.2)	1.38 (0.93–2.04)	0.11	1.39 (0.91–2.12)	0.13
MEG3 rs4081134						
GG	392 (58.7)	310 (60.9)	1.00		1.00	
AG	230 (34.4)	167 (32.8)	0.92 (0.72–1.18)	0.50	0.87 (0.67–1.14)	0.31
AA	46 (6.9)	32 (6.3)	0.88 (0.55–1.41)	0.60	0.82 (0.49–1.37)	0.44
Dominant model	276 (41.3)	199 (39.1)	0.91 (0.72–1.15)	0.44	0.86 (0.67–1.11)	0.25
Recessive model	622 (93.1)	477 (93.7)	0.91 (0.57–1.45)	0.68	0.88 (0.53–1.45)	0.61
miR-181b rs322931						
CC	473 (70.8)	315 (61.9)	1.00		1.00	
CT	179 (26.8)	170 (33.4)	1.43 (1.11–1.84)	0.006	1.48 (1.13–1.95)	0.005
TT	16 (2.4)	24 (4.7)	2.25 (1.18–4.31)	0.01	1.84 (0.91–3.70)	0.09
Dominant model	195 (29.2)	194 (38.1)	1.49 (1.17–1.91)	0.001	1.52 (1.17–1.97)	0.002
Recessive model	652 (97.6)	485 (95.3)	2.02 (1.06–3.84)	0.03	1.64 (0.82–3.27)	0.16

*IS* ischemic stroke, *OR* odds ratio, *CI* confidence interval. ^†^Adjusted by age, gender, hypertension, and diabetes mellitus

Stratification analyses were applied to evaluate the association between the 3 polymorphisms and clinical features of IS, such as TCH, TG, HDL-C, and LDL-C. As shown in Table 3, patients carrying miR-181b rs322931 CT/TT genotypes had higher LDL-C levels compared to those carrying CC genotype (*P* = 0.01). No evident association was found between MEG3 rs7158663 and rs4081134 and clinical features of IS.

**Table 3:** Stratification analyses of polymorphisms in MEG3/miR-181b and clinical features of IS

Genotypes	TCH (mmol/L)		TG (mmol/L)		HDL-C (mmol/L)		LDL-C (mmol/L)	
	Mean ± SD	*P* value	Mean ± SD	*P* value	Mean ± SD	*P* value	Mean ± SD	*P* value
MEG3 rs7158663								
GG	5.07 ± 0.72		1.82 ± 1.08		1.56 ± 0.38		2.74 ± 0.98	
AG/AA	5.02 ± 0.72	0.36	1.87 ± 1.12	0.63	1.58 ± 0.39	0.54	2.63 ± 0.97	0.18
MEG3 rs4081134								
GG	5.07 ± 0.66		1.84 ± 1.06		1.58 ± 0.39		2.71 ± 0.93	
AG/AA	5.00 ± 0.80	0.31	1.86 ± 1.16	0.81	1.55 ± 0.37	0.36	2.65 ± 1.05	0.49
miR-181b rs322931								
CC	5.01 ± 0.71		1.85 ± 1.09		1.58 ± 0.37		2.60 ± 0.96	
CT/TT	5.10 ± 0.72	0.17	1.84 ± 1.12	0. 92	1.56 ± 0.40	0.73	2.82 ± 1.00	0.01

*IS* ischemic stroke, *TCH* total cholesterol, *TG* triglyceride, *HDL-C* high-density lipoprotein cholesterol, *LDL-C* low-density lipoprotein cholesterol, *SD* standard deviation

The ancestral allele frequencies of the 3 SNPs based on data from the 1000 Genomes Project are summarized in Table 4. Compared with the data in the current study, the frequency of the rs7158663 G allele was significantly lower in Africans (12.8–32.5% vs. 73.8%) whereas the distributions of the rs4081134 G and rs322931 C were almost similar among the populations.

**Table 4:** The ancestral allele frequencies of the 3 SNPs based on data from the 1000 Genomes Project

Population	rs7158663 G, %	rs4081134 G, %	rs322931 C, %
HapMap-CEU	50.9	62.6	81.7
HapMap-HCB	74.4	77.9	84.9
HapMap-JPT	66.9	80.8	83.1
HapMap-YRI	12.8	77.9	84.8
HAPMAP-ASW	23.5	80.6	81.6
HAPMAP-CHB	74.4	78.1	84.2
HAPMAP-CHD	81.8	72.4	84.9
HAPMAP-GIH	69.9	63.6	79.0
HAPMAP-LWK	25.0	80.6	80.9
HAPMAP-MEX	59.0	74.0	65.3
HAPMAP-MKK	32.5	79.0	80.7
HAPMAP-TSI	52.8	65.3	88.1
Data in the current study	73.8	75.9	84.2

*CEU*, Utah residents with Northern and Western European ancestry from the CEPH collection, *HCB* Han Chinese in Beijing, China, *JPT* Japanese in Tokyo, Japan, *YRI* Yoruba in Ibadan, Nigeria, *ASW* African ancestry in Southwest USA, *CHB* Han Chinese in Beijing, China, *CHD* Chinese in Metropolitan Denver, Colorado, *GIH* Gujarati Indians in Houston, Texas, *LWK* Luhya in Webuye, Kenya, *MEX* Mexican ancestry in Los Angeles, California, *MKK* Maasai in Kinyawa, Kenya, *TSI* Toscans in Italy

### Combined analysis

Combined analysis was then performed to estimate the effect of rs7158663 - rs322931 and rs4081134 - rs322931 on IS risk. As shown in Tables 5, 19.3% of cases and 15.7% of controls had the combined genotypes of rs7158663 GG and rs322931 CT/TT, and carriers with these loci had a 1.62-fold increased risk of IS compared to carriers with the genotypes of rs7158663 GG and rs322931 CC (95% CI: 1.15–2.27, *P* = 0.005). Furthermore, 18.9% of cases and 13.5% of controls had the combined genotypes of rs7158663 AG/AA and rs322931 CT/TT, and carriers with these loci had a 1.85-fold increased risk of IS compared to carriers with the genotypes of rs7158663 GG and rs322931 CC (95% CI: 1.30–2.62, *P* = 0.001). Nevertheless, the distributions of combined genotypes of rs4081134 and rs322931 did not vary between cases and controls.

**Table 5:** Combined analyses of polymorphisms in MEG3/miR-181b with IS risk

Combined genotypes	Controls, *n* = 668 (%)	IS, *n* = 509 (%)	OR (95% CI)	*P* value
MEG3 rs7158663- miR-181b rs322931				
rs7158663 GG + rs322931 CC	267 (40.0)	154 (30.3)	1.00	
rs7158663 GG + rs322931 CT/TT	105 (15.7)	98 (19.3)	1.62 (1.15–2.27)	0.005
rs7158663 AG/AA + rs322931 CC	206 (30.8)	161 (31.6)	1.36 (1.02–1.80)	0.04
rs7158663 AG/AA + rs322931 CT/TT	90 (13.5)	96 (18.9)	1.85 (1.30–2.62)	0.001
MEG3 rs4081134- miR-181b rs322931				
rs4081134 GG + rs322931 CC	281 (42.1)	203 (39.9)	1.00	
rs4081134 GG + rs322931 CT/TT	111 (16.6)	107 (21.0)	1.33 (0.97–1.84)	0.08
rs4081134 AG/AA + rs322931 CC	192 (28.7)	112 (22.0)	0.81 (0.60–1.08)	0.16
rs4081134 AG/AA + rs322931 CT/TT	84 (12.6)	87 (17.1)	1.43 (1.01–2.03)	0.04

*IS* ischemic stroke, *OR* Odds ratio, *CI* confidence interval

### Haplotype analysis

Despite the absence of an LD among the 3 SNPs, the AGC and GGT haplotypes showed an increased risk of IS compared to the GGC haplotype (AGC vs. GGC: OR = 1.51, 95% CI: 1.20–1.90, *P* <  0.001; GGT vs. GGC: OR = 1.55, 95% CI: 1.16–2.07, *P* = 0.003) (Table 6).

**Table 6:** Haplotype analysis of the MEG3/miR-181b polymorphisms between cases and controls

Haplotype^a^	Controls, N (%)	IS, N (%)	OR (95% CI)	*P* value
GGC	653 (48.9)	429 (42.1)	1.00	
AGC	206 (15.4)	204 (20.0)	1.51 (1.20–1.90)	< 0.001
GAC	180 (13.5)	127 (12.5)	1.07 (0.83–1.39)	0.59
GGT	111 (8.3)	113 (11.1)	1.55 (1.16–2.07)	0.003
AAC	85 (6.4)	40 (3.9)	0.72 (0.48–1.06)	0.10
AGT	44 (3.3)	41 (4.0)	1.42 (0.91–2.21)	0.12
GAT	41 (3.1)	37 (3.6)	1.37 (0.87–2.18)	0.18

^a^Only the frequency > 3% was presented

### Multivariate regression analysis

Multivariate logistic regression analysis initially included the following variables: age, gender, hypertension, diabetes mellitus, TCH, TG, HDL-C, LDL-C, MEG3 rs7158663, rs4081134 and miR-181b rs322931. After stepwise testing, independent factors of IS were identified, including hypertension (OR = 4.97, 95%CI: 3.67–6.73, *P* <  0.001), TCH (OR = 1.24, 95%CI: 1.02–1.49, *P* = 0.03), TG (OR = 5.89, 95%CI: 4.43–7.83, *P* <  0.001), LDL-C (OR = 1.90, 95%CI: 1.61–2.24, *P* <  0.001), and rs322931 CT/TT genotypes (OR = 1.41, 95%CI: 1.04–1.91, *P* = 0.03) (Table 7).

**Table 7:** Logistic regression analysis for independent risk factors of ischemic stroke

Variables	B	Walds	OR (95% CI)	*P* value
Hypertension	1.60	107.13	4.97 (3.67–6.73)	< 0.001
TCH	0.21	4.70	1.24 (1.02–1.49)	0.03
TG	1.77	148.89	5.89 (4.43–7.83)	< 0.001
LDL-C	0.64	59.63	1.90 (1.61–2.24)	< 0.001
rs322931 CT/TT	0.34	4.82	1.41 (1.04–1.91)	0.03

*OR* odds ratio, *CI* confidence interval, *TCH* total cholesterol, *TG* triglyceride, *LDL-C* low-density lipoprotein cholesterol

### Effect of the rs322931 on transcriptional activity

**Fig. 2:**
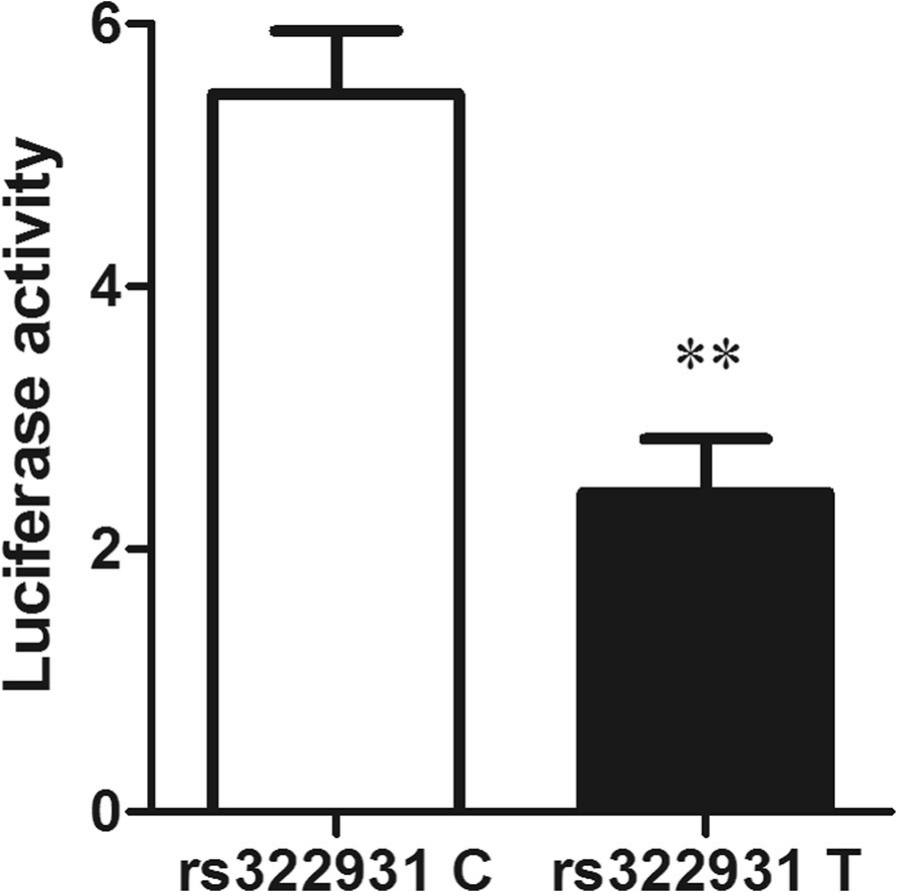
Effect of the rs322931 on transcriptional activity. The fragment containing the rs322931 C or rs322931 T allele was transfected into HEK293 cells. pGL3-SV40 was used as an internal control and relative luciferase activity was measured 48 h post-transfection. Data are expressed as mean ± standard error (** *P* <  0.01)

To elucidate the effect of the rs322931 on transcriptional activity, we performed a luciferase reporter assay and found that HEK293 cells transfected with the rs322931 T allele had lower levels of luciferase activity than cells transfected with the rs322931 C allele (*P* <  0.01) (Fig. 2), suggesting that decreased transcriptional activity mediated the risk effect of the rs322931 T allele.

## Discussion

In the current study, we aimed to investigate the association of genetic variants in MEG3/miR-181b with susceptibility to IS. We found a significant difference in genotypic distribution of miR-181b rs322931 and IS. Compared to miR-181b rs322931 TT genotype, CT and CT/TT genotypes were found to confer 1.48- and 1.52-fold increased risks of IS. Stratification analyses showed that patients carrying miR-181b rs322931 CT/TT genotypes had higher LDL-C levels. Although we did not observe any significant association between MEG3 rs7158663 and IS, the combined genotypes of rs7158663 GG and rs322931 CT/TT were associated with a 62% increased risk of IS. Multivariate logistic regression analysis identified that rs322931 CT/TT genotype was a risk factor for IS in addition to hypertension, TCH, TG, and LDL-C. Further dual-luciferase reporter assay showed that the rs322931 T allele had lower levels of luciferase activity than the rs322931 C allele. Our results indicate that miR-181b rs322931 may singly or jointly contribute to the risk of IS in the Chinese population.

Previous association studies focused on SNPs in the protein-coding genes and the risk of IS [16, 17]. For example, Bao et al. reported an SNP rs6265 AA genotype in *BDNF* had a 0.57-fold decreased risk of IS [16]. Chen et al. reported another SNP *ALOX5AP* SG13S114 A allele had a 0.86-fold decreased risk of IS [17]. Recently, the role of non-coding RNAs in the pathogenesis of IS attracted much attention, with differentially expressed lncRNAs in both human and animal model of IS [22, 23]. Moreover, lncRNA-related SNPs were reported to be potential genetic markers and therapeutic targets of IS. Wang et al. investigated 6 tagSNPs in lncRNA H19 and found that rs217727 TT and rs4929984 AA genotype increased IS risk, with an adjusted OR of 4.29 and 3.02, respectively [40]. The findings indicate that lncRNAs play fundamental roles in the development of IS.

MEG3, located on chromosome 14q32.3 in human genome, was firstly identified as a lncRNA with function of tumor suppressor [41]. Subsequent results revealed that MEG3 is implicated in biological processes of IS [26–28, 30]. Altered expression of cerebral MEG3 was observed both in vitro and in vivo, activating p53 to mediate ischemic neuronal death in stroke [27, 28]. Downregulation of MEG3 can protect against ischemic damage and enhance neurobehavioral outcomes [26, 30]. Furthermore, MEG3 functions as a competing endogenous RNA for miR-181b to regulate 12/15-LOX expression in middle cerebral artery occlusion-induced ischemic infarct of brain nerve cells [29]. In 2016, an association study including 518 cases and 527 controls was conducted by Cao and colleagues who genotyped 5 tagSNPs in MEG3 and found that rs7158663 AA genotype but not other SNPs increased the risk of colorectal cancer [33]. In 2018, Zhou et al. genotyped 2 tagSNPs in MEG3 in 392 neuroblastoma children and 783 controls and found that subjects carrying rs4081134 AG/AA genotypes tended to develop neuroblastoma among subgroups with age > 18 month and clinical stage III + IV disease, with an adjusted OR of 1.36 and 1.47, respectively [34]. To date, we are not aware of any association study of SNPs in MEG3 in IS. In this study, we explored for the first time the association between 2 tagSNPs (i.e., rs7158663 and rs4081134) and IS risk in 509 IS patients and 668 controls. Although we failed to find any association of the 2 SNPs with IS risk in single-site analysis, the combined genotypes of MEG3 rs7158663 and miR-181b rs322931 conferred the susceptibility of IS, further supporting the idea that MEG3 and miR-181b have a crosstalk in the pathophysiology of IS [29].

miR-181b, a kind of miRNAs, was demonstrated to be a key player in the regulation of circulating low-density lipoprotein cholesterol levels [42] and neural ischemic injuries [43–47]. In mouse brain following middle cerebral artery occlusion and oxygen–glucose deprivation-treated cells, miR-181b was observed to be downregulated, and the downregulation protects against ischemic injury via targeting heat shock protein A5, ubiquitin carboxyl-terminal hydrolase isozyme L1 and cylindromatosis [43, 47]. Administration of miR-181b can not only modulate macrophage polarization and attenuate atherosclerotic plaque vulnerability but also increase M2 markers in macrophage cells through directly targeting Notch1 [44]. Exosomes-derived miR-181b-5p can accelerate angiogenesis of ischemic injury both in vivo and in vitro by targeting transient receptor potential melastatin 7 [45]. Moreover, electroacupuncture can enhance rehabilitation against stroke by targeting miR-181b/PirB/ RhoA/GAP43 axis and leading to epigenetic changes [46]. These findings strongly suggest that miR-181b may be a potential therapeutic target for IS. No study however investigated the association of miR-181b related SNP with IS risk. In this study, we hypothesized that rs322931 may contribute to the risk of IS since the polymorphism can influence the expression of miR-181b [35]. Our findings for the first time confirmed this hypothesis. We found that miR-181b rs322931 CT/TT was an independent risk factor in both single-site association analysis and logistic regression analysis. The risk effect may partly be caused by lower levels of transcriptional activity.

Some limitations in this study should be noted. The study is based on hospitalized cases and controls, and thus the selection bias cannot be ruled out. Considering some demographic and clinical factors influencing genetic predisposition of IS, it would be interesting to perform gene-environment interaction analysis. However, lack of comprehensive data, such as drinking habits and tobacco smoking makes it unfeasible to run such an analysis. We, therefore, encourage further investigation of MEG3 rs7158663, rs4081134 and miR-181b rs322931 in population-based studies and gene-environment interaction analysis, which may reveal reliable models of the SNPs in IS occurrence.

## Conclusion

To our knowledge, this is the first study analyzing genetic polymorphisms in MEG3/miR-181b with IS to date, and it indicates a significant association between miR-181b rs322931 and the risk of IS, partly caused by reducing transcriptional activity. Additionally, this study adds novel information about combined genotypes (rs7158663 GG + rs322931 CT/TT and rs7158663 AG/AA + rs322931 CT/TT) increasing IS risk and rs322931 CT/TT genotype being an independent risk factor for IS. Further investigation is required regarding how the polymorphism influences IS risk.

## Abbreviations

*ALOX5AP*: Arachidonate 5-lipoxygenase-activating protein
ASW: African ancestry in Southwest USA
*BDNF*: Brain-derived neurotrophic factor
CEU: Utah residents with Northern and Western European ancestry from the CEPH collection
CHB: Han Chinese in Beijing, China
CHD: Chinese in Metropolitan Denver, Colorado
CIs: Confidence intervals
GIH: Gujarati Indians in Houston, Texas
HCB: Han Chinese in Beijing, China
HDL-C: High-density lipoprotein cholesterol
HWE: Hardy Weinberg equilibrium
IS: Ischemic stroke
JPT: Japanese in Tokyo, Japan
LD: Linkage disequilibrium
LDL-C: low-density lipoprotein cholesterol
lncRNAs: Long non-coding RNAs
LWK: Luhya in Webuye, Kenya
MEG3: Maternally expressed gene 3
MEX: Mexican ancestry in Los Angeles, California
miRNAs: microRNAs
MKK: Maasai in Kinyawa, Kenya
OR: odds ratio
SNPs: single nucleotide polymorphisms
TBP: TATA bingding protein GR Glucocorticoid receptor
TCH: Total cholesterol
TG: Triglyceride
TSI: Toscans in Italy
YRI: Yoruba in Ibadan, Nigeria
